# GTASynth: 3D synthetic data of outdoor non-urban environments.

**DOI:** 10.1016/j.dib.2022.108412

**Published:** 2022-06-22

**Authors:** Giovanni Curnis, Simone Fontana, Domenico G. Sorrenti

**Affiliations:** Dipartimento di Informatica, Sistemistica e Comunicazione, Università degli Studi di Milano - Bicocca, Italy

**Keywords:** Synthetic Dataset, Grand theft Auto 5, Point Cloud, Point Cloud Registration, Robotics, 3D Data, SLAM, mapping

## Abstract

Developing point clouds registration, SLAM or place recognition algorithms requires data with a high quality ground truth (usually composed of a position and orientation). Moreover, many machine learning algorithms require large amounts of data for training. However, acquiring this kind of data in non-urban outdoor environments poses several challenges. First of all, off-road robots are usually very expensive. Above all, producing an accurate ground truth is problematic. Even the best sensor available, *i.e.* RTK GPS, cannot guarantee the required accuracy in every condition. Hence the scarcity of this kind of dataset for point clouds registration or SLAM in off-road conditions.

For these reasons, we propose a synthetic dataset generated using Grand Theft Auto V (GTAV), a video game that accurately simulates sensing in outdoor environments. The data production technique is based on DeepGTAV-PreSIL [1]: a simulated LiDAR and a camera are installed on a vehicle which is driven through the GTAV map. Since one of the goals of our work is to produce a large amount of data to train neural networks which will then be used with real data, we have chosen the characteristics of the sensors to accurately simulate real ones.

The proposed dataset is composed of 16.207 point clouds and images, divided into five sequences representing different environments, such as fields, woods and mountains. For each pair of point clouds and images we also provide the ground truth pose of the vehicle at the acquisition.

## Specifications Table

**Table:** 

Subject	Computer Vision and Pattern Recognition
Specific subject area	3D Perception and mapping
Type of data	Image (*.png)Text (*.txt)Pointcloud (*.pcd, *.bin)
How the data were acquired	The data has been produced using Grand Theft Auto V, along with the DeepGTAV-PreSIL framework [1]. The Images were saved in high quality PNG format with a dimension of 1920 × 1080 pixels, while the point clouds use the PCD format [2] and have been obtained using a virtual LiDAR.LiDAR specifications (modeled after the Velodyne HDL-64E): • 64 beams • Vertical FoV of 26.9° (+8° up and -18.9°down) • Vertical resolution of 0.420° • Horizontal FoV of 90° • Horizontal resolution of 0.09° Camera specifications: • Vertical FoV of 59° • Horizontal FoV of 90° • Output of 1920 × 1080 pixels
Data format	Mixed (raw and processed)
Description of data collection	The data has been collected using a virtual LiDAR and a virtual camera mounted on a vehicle in the Grand Theft Auto V game. The vehicle has been driven through the game map, to collect data representing different kinds of non-urban outdoor environments.
Data source location	The data were collected in a virtual city in the game Grand Theft Auto V. The city was designed to be similar to Los Angeles (USA).The data were processed at: • Institution: University of Milano-Bicocca • City: Milan • Country: Italy
Data accessibility	Repository name: OSFURL: https://osf.io/pku3e/DOI: 10.17605/OSF.IO/PKU3EDataset tools are available on GitHub: https://github.com/iralabdisco/GTAV-360-Pointcloud-DatasetCode used to generate the dataset available on GitHub:https://github.com/iralabdisco/DeepGTAV-PreSIL_GTASynth
Related research article	**NONE**

## Value of the Data

• Collecting 3D datasets in a natural outdoor environment is a challenging activity. Vehicles or robots with off-road capabilities are very expensive and not widely available. A reliable ground-truth pose is essential if the data are to be used with point clouds registration or SLAM algorithms. However, the only viable solution is to use an RTK GPS, which is expensive and unreliable when the sky is occluded by vegetation, dense clouds, or when the hemisphere in view is reduced by the terrain. For these reasons, there are very few datasets of this kind available, and even fewer have a high quality ground truth, as documented by Fontana *et al.*3. We propose a solution based on high quality, realistic, synthetic point clouds, and camera images, whose ground truth pose is, of course, perfect. The dimension of our dataset is huge, compared to other existing solutions. Moreover, it depicts environments that are not covered by other datasets.
• Testing a point clouds registration or SLAM algorithm requires a large amount of data, with a reliable ground truth pose. Moreover, machine learning based algorithms require an even larger amount of data during the training phase. While there is a large availability of data aimed at autonomous driving applications, depicting urban environments, a large dataset is simply not available for natural non-urban environments, such as forests, fields or mountaints. Therefore, we think that researchers in the field of 3D perception could greatly benefit from the proposed dataset. Non exhaustive examples of applications are agricultural or search and rescue robotics.
• This dataset is particularly useful when used in conjunction with registration problems generating techniques, which randomly generate initial misalignments and chooses pairs of point clouds to cover different degrees of overlap. Examples are those used by Fontana *et al.* [3] and by Drory *et al.* [4]*.*
• This dataset can be used to train machine learning algorithms that require point clouds and/or pictures with an associated pose [5], [6], [7], [8]. The idea is to train on our large synthetic dataset and then test on real data.
• Besides the raw and processed data, we also published on GitHub the code we used to produce the datasets. The repository is a fork of the original DeepGTAV-PreSIL framework and contains instructions to reproduce the data collection process. Therefore, authors who need data with different characteristics, such as a different laser scanner, or depicting a different environment, can use the provided tools to produce another dataset with very little effort. The GitHub repository is: https://github.com/iralabdisco/DeepGTAV-PreSIL_GTASynth

## 1 Data Description

A point cloud is a set of points with 3D coordinates. These points can be used to describe the shape of a 3D object or to represent large scenes such as streets or buildings. They are widely used in robotic and remote sensing applications.

**Fig. 1:**
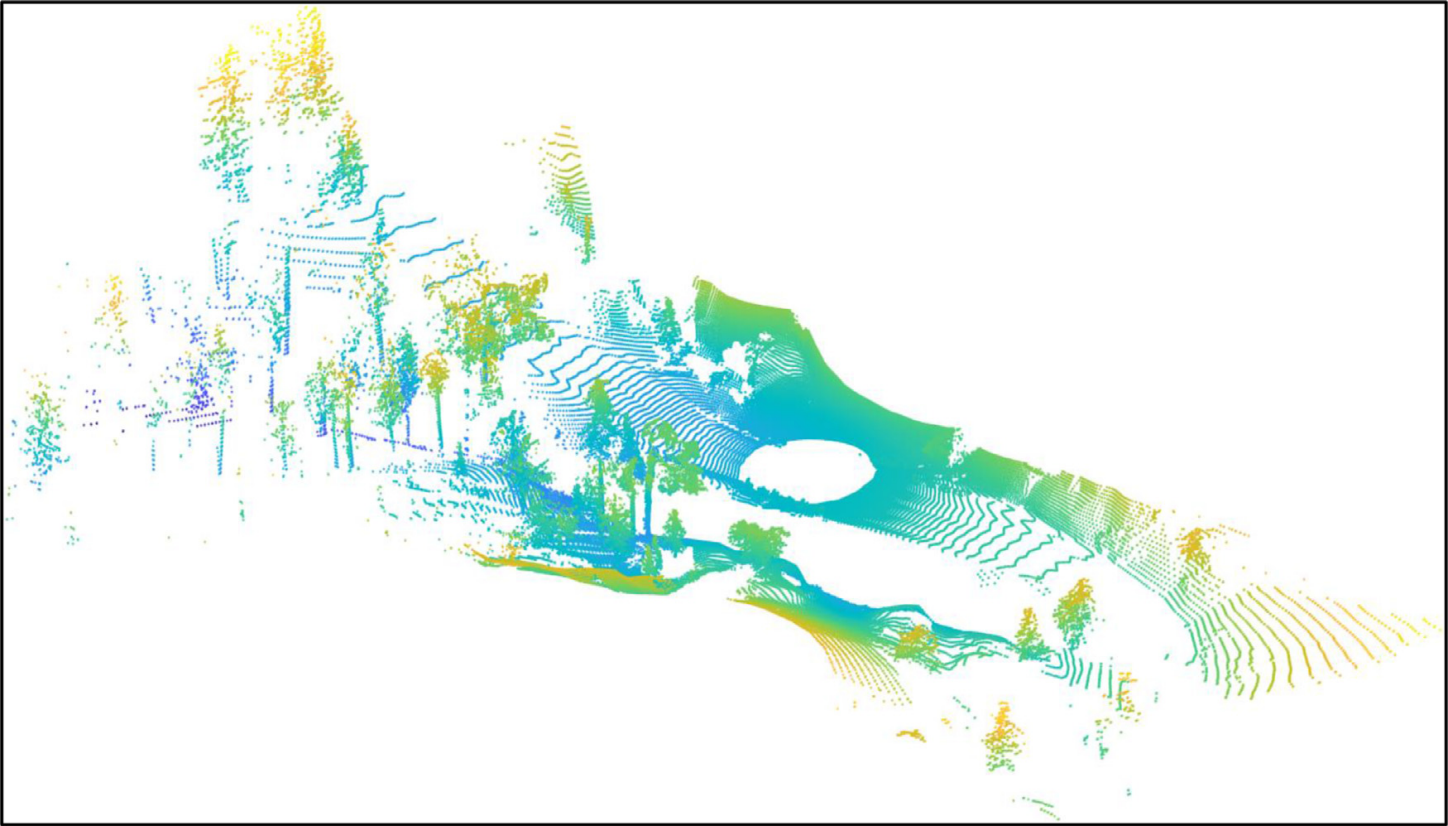
An example of point cloud in the GTASynth dataset.

**Fig. 2:**
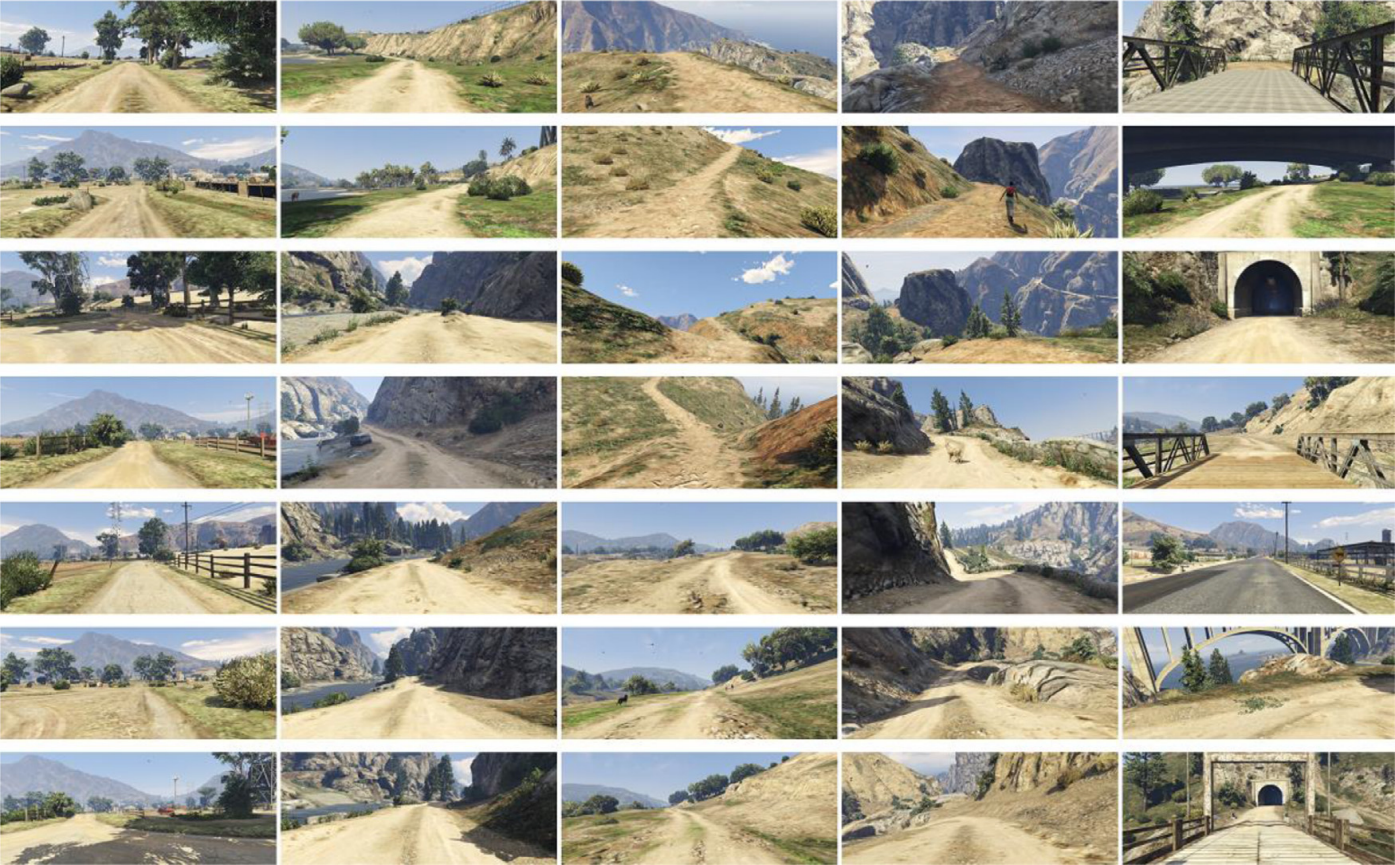
Examples of images in the GTASynth dataset.

The GTASynth dataset is composed of 16.207 pairs of synthetic point clouds and images (Figs. 1 and 2) acquired from the same point of view. They have been collected while navigating through the game map of Grand Theft Auto V, using a framework based on DeepGTAV-PreSIL [1] . The data are divided into 6 sequences representing different non-urban outdoor environments. Table 1 lists the six sequences, along with their dimension, length and whether we passed multiple times through the same places (the “Loops” column), a very important characteristic for SLAM algorithms, which usually have to detect and exploit loop closures.

**Table 1:** The sequences wich compose the GTASynth dataset.

Name	Pairs	Length (km)	Loops
river_sequence	1966	6.207	1
park_sequence	1516	2.873	6
mountain_sequence	2678	6.619	1
hill_sequence	3108	12.732	7
field_sequence	3252	7.356	8
canyon_sequence	3687	8.871	1

The GTASynth dataset can be downloaded in two versions: **raw data** and **processed data**. We suggest researchers to use the processed data; however, we also provide the raw data to better document the post-production process.

The dataset is structured as follows.

### 1.1 Raw Data

• **Velodyne**: contains four folders, one for each of the 90° sectors used to create the 360° point clouds. We collected and then merged four point clouds because the software we used cannot simulate a 360° LiDAR. Each folder contains the corresponding point clouds, named <num_frame>.bin.
• **Pose**: contains four folders, one for each of the 90° sectors used to create the 360° point cloud. Each of these folders contains files <num_frame>.txt with the pose of the lidar *w.r.t.* map reference frame. The first two rows of the file are always “*0, 0, 0*”, the third row is the position of the lidar and the camera *w.r.t* the map reference frame, the 4th row is the rotation of the LiDAR, while the last row is the rotation of the camera. As can be seen, the camera and the LiDAR are in the same position, but have different orientations. The rotations are expressed as roll, pitch and yaw angles in degrees.

### 1.2 Processed Data

• **location_360**: contains the ground truth poses of the LiDAR *w.r.t.* the map reference frame. Files are named <num_frame>.txt.
• **velodyne_360**: the 360° point clouds, named <num_frame>.pcd.
• **Image**: front camera images saved as <num_frame>.png.
• **location.txt**: id, position and orientation of the camera for each captured frame.
• **calib.txt:** contains the rotation of the camera, expressed by a rotation matrix, *w.r.t.* the LiDAR and the intrinsic projection matrix of the camera. Both matrices have been flattened in row-major format.
• **<sequence_name>_complete_map.pcd**: the map of the scanned environment. It is composed of all the point clouds of the sequenced, downsampled with a voxel grid with a leaf-size of 1 meter.

### 1.3 Dataset Tools

**Fig. 3:**
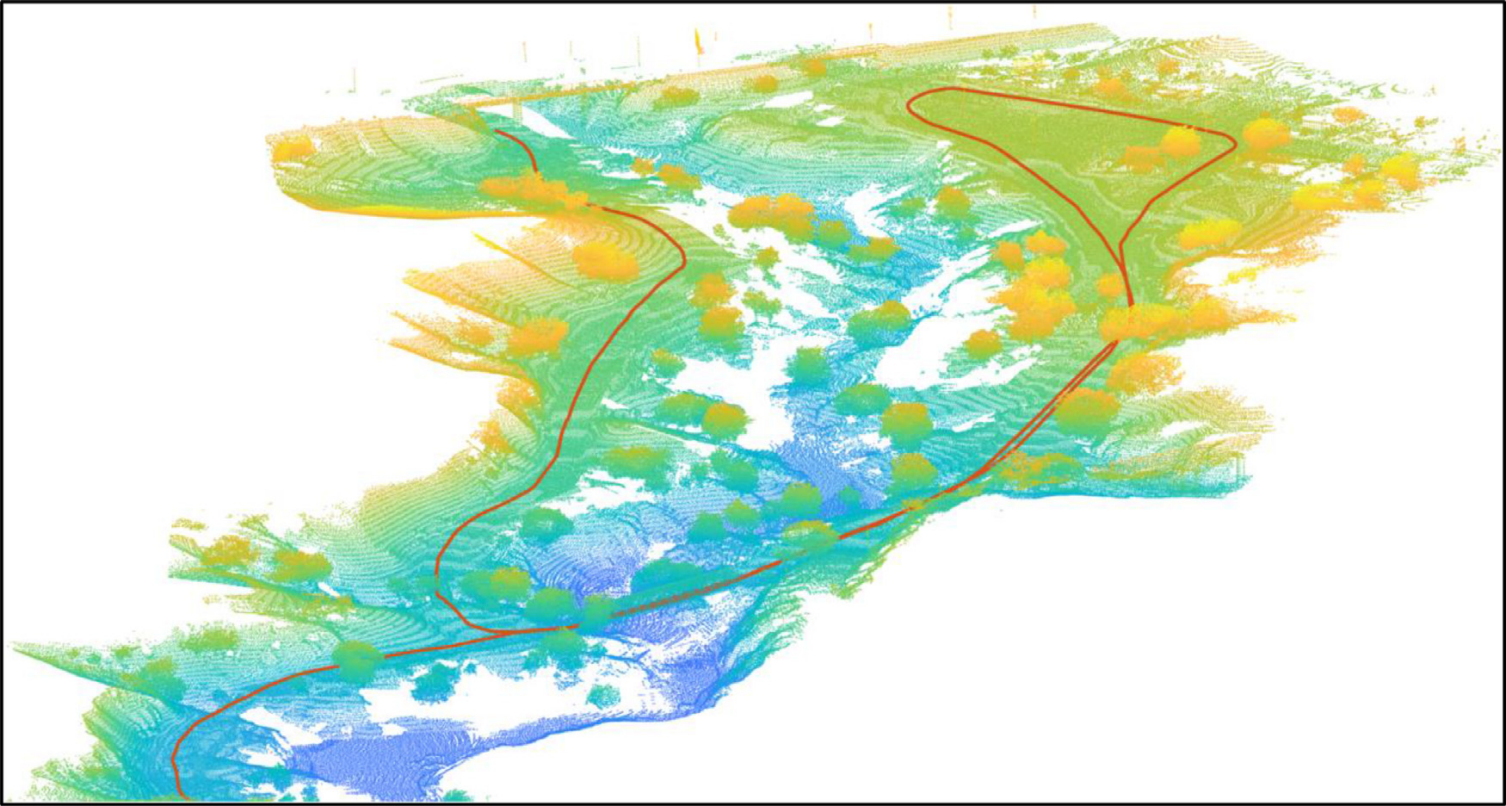
An example of map and path of the vehicle in the GTASynth dataset.
• **helperFrameLocationOnMap.m:** view a single point cloud on the map.[MATLAB]

• **create_360_from _velofile.py:** takes as input four raw point clouds to produce the 360° view. [Python]
• **create_map.py:** creates a map of the environment using the processed data. [Python]
• **point_cloud_player.py:** reproduce the point clouds in sequence, as they were acquired. [Python]
• **tools.py:** contains methods to read the data in the dataset. [Python]
• **helperPointCloudPlayer.m:** the same as **Point_cloud_player.py**. [MATLAB]
• **helperMapPathVisualizer.m:** with the path followed by the vehicle on the map. [MATLAB] (Fig. 3)

## 2 Experimental Design, Materials and Methods

To collect the data, we used Grand Theft Auto V, along with a framework developed to exploit the game as a computer vision research environment, DeepGTAV-PreSIL [1,9,10].

We drove a simulated vehicle through various parts of the game map and collected point clouds and RGB images at a fixed frequency of 10Hz. The chosen acquisition frequency is a trade-off between frame rate and size of the dataset. A higher acquisition frequency would make the dataset overly large, without, in our opinion, many advantages. Indeed, 10Hz is enough for most SLAM, point clouds registration or place recognition applications. On the other hand, the dataset is probably not suited to high-frequency applications such as visual odometry.

The framework we used allows the collection of 3D data with a maximum horizontal field of view (FoV) of 90°. However, most LiDARS have a FoV of 360° or slightly less. We wanted our synthetic data to be as similar as possible to real data. Therefore, we divided the area surrounding the vehicle in four sectors, each corresponding to a FoV of 90°. Eventually, at each pose we collected four point clouds and merged them in post-production.

To obtain realistic data, we simulated a LiDAR similar to the very popular Velodyne HDL-64E. It had a vertical FoV of 26.9° (+8° up and -18.9° down) with 0.420° of vertical angular resolution. The horizontal FoV was 90° with a resolution of 0.09°, and a maximum range of 100 meters.

For each 360° point cloud, we also collected an image obtained using a virtual camera with a vertical FoV of 59° and an horizontal FoV of 90°. We would prefer a dataset composed of images produced with multiple cameras or with a 360° camera, similarly to the nuScenes [11] or the nuPlan [12] datasets. However, this was not possible because of technical limitations of the framework we used. Nevertheless, the setup we simulated is very similar to the popular KITTI dataset [13].

The result is a large dataset composed of synthetic point clouds and images, with known poses, collected as if the sensors were mounted on a vehicle traversing a non-urban outdoor environment. To document and make the data creation process reproducible, we published the tools we used on a GitHub repository: https://github.com/iralabdisco/GTAV-360-Pointcloud-Dataset. Moreover, in a fork of the original DeepGTAV-PreSIL framework, we provide the code that can be used to produce other datasets, with different characteristics or representing different environments from those in the GTAV game map. The link to this repository is: https://github.com/iralabdisco/DeepGTAV-PreSIL_GTASynth

## Ethics Statements

This research did not involve any human subjects or animal experimentation.

## CRediT Author Statement

**Giovanni Curnis:** Conceptualization, Data curation, Software, Writing - original draft. **Simone Fontana:** Conceptualization, Supervision, Writing - Reviewing and Editing. **Domenico G. Sorrenti:** Writing - Reviewing and Editing.

## Declaration of Competing Interest

The authors declare that they have no known competing financial interests or personal relationships that could have appeared to influence the work reported in this paper.
